# More folate intake, less suicide attempts: is there a link?

**DOI:** 10.1192/j.eurpsy.2023.2369

**Published:** 2023-07-19

**Authors:** N. Kouki, A. Maamri, M. A. Zaafrane, D. Bougacha, A. Hajri, H. Zalila

**Affiliations:** 1 Outpatient department, Razi hospital, Manouba, Tunisia

## Abstract

**Introduction:**

Suicide is a major health problem, mostly related to mental health disorders. However psychological autopsies have revealed the presence of unexpected suicide factors such as dietary patterns particularly folate intake. Which is a naturally occurring form of B9 essential for neurogenesis, nucleotide synthesis and methylation of homocysteine.

**Objectives:**

The aim of our study is to identify the link between folate intake and suicidality.

**Methods:**

Our literature review was based on the PubMed interface and adapted for 2 databases: Science Direct and Google Scholar using the following combination ( suicide [MeSH terms]) AND (folate [MeSH terms]) AND (prevention [MeSH terms]).

**Results:**

Stress-induced neuronal dysfunctions interact with genetic and environmental factors, including diet, to precipitate mental health disorders in vulnerable or predisposed individuals especially mood disorders.

In one hand, studies showed delayed onset of clinical improvement even treatment resistance in depressive patients treated with fluoxetine associated with low folate levels.

In the other hand Folate depletion has been linked to serotoninergic metabolism disturb.

Moreover, co-administration of methylfolate, a highly absorbable form of folic acid, has been found to augment the effects of SSRIs .A duration-response analysis (1-mg dosage) revealed a 5% decrease in suicidal events per month of additional treatment.

**Conclusions:**

Accumulating data have shown that these nutrients can enhance neurocognitive function, and may have therapeutic benefits for depression and suicidal behaviors . But the pathological mechanism remains unclear that’s why further studies are needed for a better comprehension and efficiency.

**Disclosure of Interest:**

None Declared

